# Telesupervision in Psychotherapy: A Bibliometric and Systematic Review

**DOI:** 10.3390/ijerph192316366

**Published:** 2022-12-06

**Authors:** Paola Andreucci-Annunziata, Augusto Mellado, Alejandro Vega-Muñoz

**Affiliations:** 1 Instituto de Investigación y Postgrado, Facultad de Ciencias de la Salud, Universidad Central de Chile, Santiago 8330507, Chile; 2 Escuela de Doctorado, Universidad Rovira i Virgili, 43007 Tarragona, Spain

**Keywords:** digital supervision, clinical psychology, wellbeing, qualitative studies, quantitative studies, new technologies

## Abstract

(1) Background: This systematic review supported by a bibliometric analysis identified quantitative and qualitative empirical studies that allowed us to respond to the objective of identifying and discussing the scope and limitations of the clinical-psychotherapeutic supervision in virtual modality or telesupervision. (2) Methods: The articles were selected according to the Systematic Reviews and Meta-Analyses (PRISMA) guidelines, and the eligibility criteria proposed by the PICOS strategy (population, interventions, comparators, outcomes, and study design) based on 396 records of scientifically identified articles in the Journal Citation Report databases of the Web of Science. (3) Results: The literature review stages allowed the selection of three articles, which were added three others that were already included in a previous review, to enrich the analysis and discussion. The results of the present review highlighted aspects of nonverbal communication, alliance, comfort, preference, trust, and construction of professional identity, among others, both considering only the telesupervision format and comparing it with traditional face-to-face supervision. (4) Conclusions: The contributions that these results are providing to the understanding of the scope and limitations of the practice of telesupervision are discussed, also considering its interference in the construction of the professional identity of supervisors and supervisees.

## 1. Introduction

Clinical supervision as a training process for psychotherapists is a complex activity that has been discussed in the psychology literature [1,2]. Although its clinical-pedagogical usefulness in the development of psychotherapeutic skills, abilities, and competencies has been recognized for several decades [3,4,5], there are still doubts about its real incidence in achieving the planned therapeutic objectives with patients, especially in current times when the SARS-CoV-2 pandemic has forced us to employ virtual technologies for its implementation, clinical training, monitoring, evaluation, and feedback [6,7,8].

Research in this area has focused on the requirements of the supervisory process: identity formation, disciplinary content, transferential, and working alliance process involved in its pedagogical character pointing toward meta-theoretical commonalities [9,10,11,12,13,14]. It has also characterized: (1) the roles, functions, and activities to be developed by supervisors and supervisees in dyadic and group processes; (2) the expected outcomes in professional training; and (3) the implications for supervised patients in each instance [15,16,17,18,19].

Reconceptualizing clinical-psychotherapeutic supervision, it is possible to delimit four key dimensions that compose it: (1) a political dimension that governs it and focuses on the positioning of disciplinary knowledge and power in the agents; (2) a strategic dimension that is oriented by the former and emphasizes a certain trajectory or orientation according to the influence achieved by supervision; (3) a reflective dimension that attends to practices and questions them ethically and conceptually; and (4) a relational dimension that translates into working alliance or therapeutic alliance and that gives it existence, meaning and continuity in its condition as a network favoring exchanges, agreements, and dissent [20,21,22,23].

It is questioned how these supervision dimensions could be presented in the virtual supervision modality [24] or tele-supervision. The relational dimension of work in supervision involving supervisors and their supervisees [25] could be the most engaged with device modifications from face-to-face to virtuality [26]. This dimension involves the articulation of instructional, normative, strategic, and dialogue areas between the agents involved with the properly reflective and dialogic ones that allude to the dynamic positions or subjective positionings [21,27], which can be blurred, closed, or mutated in the contextual absence of the face-to-face that the synchronous face-to-face process would merit [28,29].

The aim of this bibliometric and systematic review is to identify and discuss the scope and limitations of telesupervision in psychotherapy, which has been used in the last decade [30,31] and during the COVID-19 pandemic [32,33]. Psychotherapists will then be able to consider the challenges of including new information technologies in their supervisory practice. It takes distance from other recent systematic reviews that have focused on answering questions about: (1) the common clinical supervision factors for psychotherapists [12]; (2) the clinical supervision effects on supervisees and patients from a certain theoretical-clinical perspective [17]; (3) the suggestions arising from empirical studies in clinical supervision with standardized guidelines [34], among the most relevant ones.

## 2. Materials and Methods

In this review, the Preferred Reporting Items for Systematic Reviews and Meta-Analyses (PRISMA) guidelines [35,36] were used, and the PICOS (participants, interventions, comparators, outcomes, and study design) strategy was used to establish the eligibility criteria for the articles [37]. According to the checklist of the PRISMA guidelines, the following quality steps for systematic reviews were verified according to the following items: 1 (title), 2 (structured abstract), 3 (rationale), 4 (objectives), 5 (eligibility criteria), 6 (sources of information), 7 (search strategy), 8 (selection process), 9 (data extraction process), 10a and 10b (data items), 11 (study risk of bias assessment), 16a and 16b (study selection), 17 (study characteristics), 18 (risk of bias in studies), 19 (results of individual studies), 23 (discussion), 25 (support), and 27 (availability of data, code and other materials). The following items were excluded from the PRISMA guidelines due to their non-applicability to the objectives of this review: 12 (effect measures), 13 (methods of synthesis), 14 (reporting bias assessment), 15 (certainty assessment), 20 (results of syntheses), 21 (reporting biases), 22 (certainty of evidence), 24 (registration and protocol), and 26 (competing interests). In addition, the initial search for articles was performed using bibliometric procedures [38].

A set of articles was used as a homogeneous citation base, avoiding the impossibility of comparing indexing databases that use different calculation bases to determine journals’ impact factors and quartiles [39,40,41,42,43], relying on the Web of Science (WoS) core collection, selecting articles published in journals indexed by WoS in the Science Citation Index Expanded (WoS-SCIE) and Social Science Citation Index (WoS-SSCI), from a search vector on supervision in psychoterapy TS = (supervis* AND psychotherap*), without restricted temporal parameters, performing the extraction on 31 August 2022. The following types of documents were included: articles, meeting abstract, review, editorial material, book review, and letter.

A complementary bibliometric analysis was carried out on a set article obtained for the topic under study. Using the fundamental bibliometric laws:

(1) Publications concentration in journals or Bradford’s Law, distributing the journals in thirds according to the decreasing number of documents published in them, establishing as the nucleus of journals with the highest concentration that cover at least 33% of the total publications [44,45].

(2) Exponential science growth or Price’s Law, through the exponential adjustment degree of the annual growth of publications, as a measure of a strong interest among the scientific community to develop studies on physical literacy, conforming a critical researcher mass developing this knowledge topic [46,47], and determining the time median and its contemporary and obsolete periods.

(3) Keyword concentration or Zipf’s Law, highlighting the most used keywords in the article set [48].

Finally, VOSviewer software version 1.6.18 (Centre for Science and Technology Studies, Leiden University, Leiden, The Netherlands) was used to perform the processing and visualization of the dataset, as well as co-occurrence, performing a fragmentation analysis with clustered visualization outputs [49,50].

Through PRISMA guidelines, the selection of articles was specified based on eligibility criteria: the target population (participants), the interventions (methodological techniques), the elements of comparison of these studies, the outcomes of these studies, and the study designs (the criteria of the PICOS strategy as shown in Table 1).

## 3. Results

**Figure 1:**
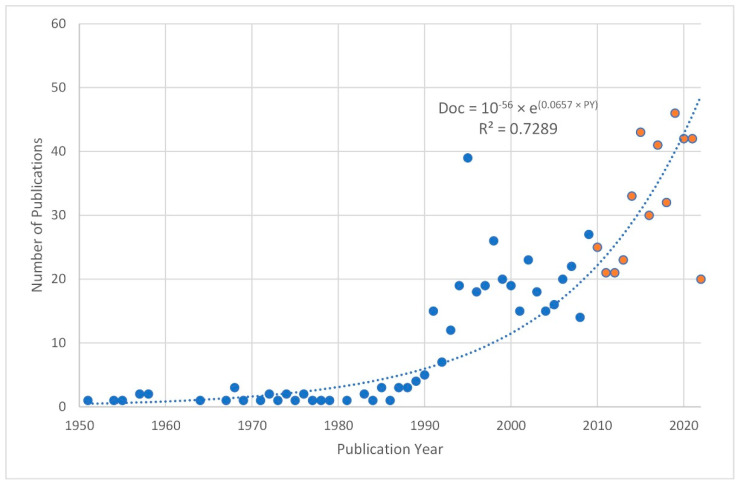
Publications on supervision in psychotherapy between 1950 and 2022. Blue dots, non-contemporary documents in data series; orange dots, contemporary data series; dashed line, exponential data series trend.

**Figure 2:**
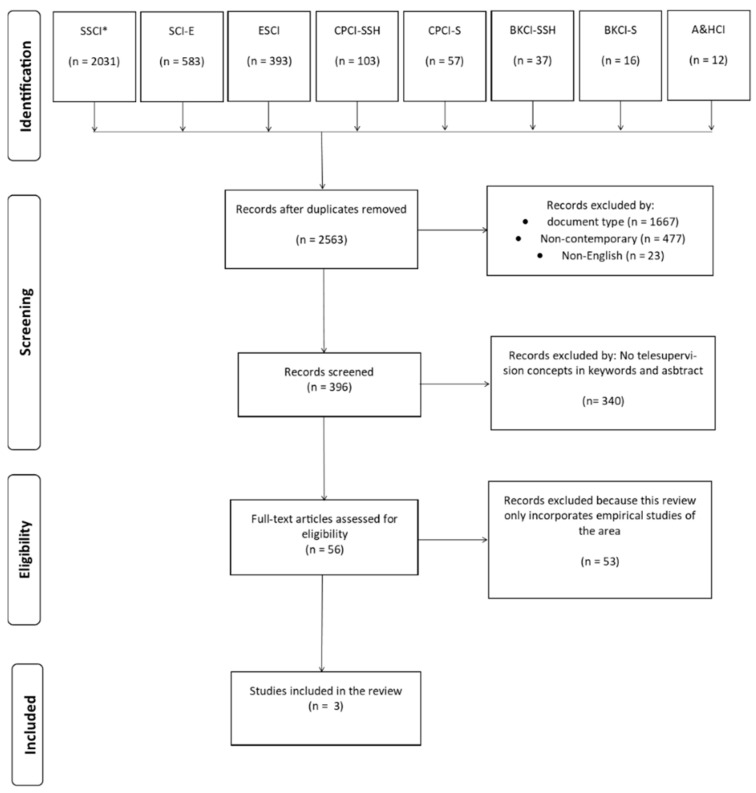
Preferred Reporting Items for Systematic Reviews and Meta-Analyses (PRISMA) analysis flow. SSCI* = Social Sciences Citation Index; SCI-EXPANDED = Science Citation Index Expanded; ESCI = Emerging Sources Citation Index; CPCI-SSH = Conference Proceedings Citation Index—Social Science & Humanities; CPCI-S = Conference Proceedings Citation Index—Science; BKCI-SSH = Book Citation Index—Social Sciences & Humanities; A&HCI = Arts & Humanities Citation Index.

The bibliometric search of articles identified a total of 2563 no repeated articles from seven different databases of the Web of Science Core Collection (i.e., SSCI; SCI-EXPANDED; ESCI; CPCI-SSH; CPCI-S; BKCI-SSH; A&HCI). Excluding records according to document type (1667) (see Table 2), non-contemporary article (blue dots) (see Figure 1) (477), and non-English-language articles (23) resulted in 396 records for screening (details in  Supplementary File S1). In addition, 340 articles not related to telesupervision keywords (virtual; ICT; Internet; computer; digital; distance; telesupervision; videoconference; telehealth; and online) both in the keywords and in the abstract were excluded, reducing the corpus analyzed to 56 full-text articles in English retrieved and screened using the selection criteria defined with the PICOS strategy. Finally, in this phase, articles that presented empirical studies not directly related to telesupervision, theoretical proposals, and theoretical-practical models of supervision but without evidence associated with a rigorous research design were excluded. The screening thus identified three articles that met the inclusion criteria as shown in Figure 2.

Using the PRISMA method, three articles were selected [51,52,53] (see Figure 2). One of these three articles [52] included, as a reference, a review of telesupervision in diverse disciplines [54], from which three other articles corresponding to empirical studies of telesupervision in psychotherapy were extracted. In this way, it was possible to enrich the systematization of the present topic with literature that had already been identified and whose quality and conclusions could be evaluated in this review.

A summary of the characteristics of the studies included in this review can be seen in Table 3.

Moreover, a quality assessment of the studies was performed (see Table 4) following the criteria proposed in the Mixed Methods Appraisal Tool (MMAT) [58].

In general, the selected studies adhere to different methodological designs, including mixed designs (qualitative and quantitative stages), and present good quality in their implementation and results, except for some limitations in the generalization of results and inaccuracies in the way two different methodologies are made to converge, in addition to presenting the material qualitatively analyzed. Aspects that could be improved in future similar research.

The narrative synthesis of the selected studies made it possible to answer the proposed research questions. For this purpose, the guide for conducting narrative syntheses in systematic reviews [59], suggested by the PRISMA-P 2015 document [60], was consulted.

The studies included among their participants psychologists, counselors, psychology counselors, and psychiatrists with different levels of training, both as supervisors and supervisees. Their methodologies and results were also varied and aimed at different dimensions of supervision work. Although it was possible to analyze them by grouping subsets of data, this review was not intended as a meta-analysis.

The 6 articles reviewed included 5 studies with supervised psychologists (two studies, 28 participants), supervisors-in-training (one study, 15 participants), supervision dyads consisting of psychiatric residents and supervisors (one study, 8 participants), and psychologists-in-training and their supervisors (one study, 16 participants). Considering the five studies, two of them used quantitative descriptive and qualitative methodology respectively, and three used mixed methods. 

The techniques used were self-report questionnaires (and one study included a questionnaire completed by trained observers) with Likert-type items in the descriptive quantitative designs and questionnaires with open-ended questions, open-ended interviews, and semi-structured interviews in the qualitative designs.

Three studies described similarities in both supervision modalities, indicating that their participants expressed positive attitudes, considering them effective, building strong relationships, possibility of development and task accomplishment [52], and evidenced no significant differences in job satisfaction and alliance [53]. The third study reported that supervisors found no differences in communication, alliance, and discomfort (frustration and displeasure) with the devices, and that external observers did not find differences in the mutuality of supervisor-supervisee contact in the two conditions [56].

Perry’s study [57] evidenced that both supervisors and supervisees experienced telesupervision as an effective means of professional identity development, although in this study, there was only comparative reference to face-to-face supervision of other supervisees (whose direct experience was not included by design), both from the perspective of supervisors and supervisees. However, in the research by Blackman et al. [51], an inverse association was found between the greater electronic safety awareness of participating psychotherapists and their comfort in sharing video or audio of consenting patients to be used as clinical work material in telesupervision.

In terms of the differences found by participants between face-to-face supervision and telesupervision, four studies point out that there are certain difficulties that telesupervision would have under such a comparison. Some supervisees noted that they found the quality of face-to-face supervision to be higher although they would participate again in the telesupervision format [52] and their preference for face-to-face supervision [53]. Both supervisors and supervisees expressed difficulties with perception and a reduction in nonverbal cues in telesupervision, as well as effects on spontaneity, expression of personal emotions, and social experience [53,55]. Some supervisees experienced face-to-face supervision as the most favorable, especially about situations that produced discontent and frustration [56], whereas some supervisors felt that by not having a face-to-face encounter in telesupervision, there is no clarity on what the supervised are meaning in the process [57].

In one of the studies [55,56], both supervisors and supervisees acknowledge that there are positive effects of telesupervision that can also be limitations. They noted that this format led them to need more thorough preparation and greater self-discipline. Some supervisees reported feeling more exposed and vulnerable, although this progressively decreased as they became more accustomed to the technology, as well as presenting themselves more freely or in a more intellectual and neutral manner. The supervisors in this study emphasized the pedagogical potential of interspersed supervision in both formats.

## 4. Discussion

This bibliometric and systematic review, guided by the PRISMA guidelines, aimed to identify and discuss the scope and limitations of the clinical-psychotherapeutic supervision in virtual, digital modality, or telesupervision. For this purpose, it had two stages of analysis, the first one being a bibliometric phase focused on WoS databases and which allowed focusing the search according to the type, publication trend and language of the eligible articles and, the second, a phase oriented to the final selection of divulgation articles of quantitative and qualitative empirical studies. In addition, to enrich the analysis, three articles on this topic, found outside the first stage and which had already been covered by a much more general previous review [54] that also included disciplines such as therapeutic counseling, educational, rehabilitation supervision, social work, psychiatry, etc., were added to enrich the analysis.

Regarding this specific topic, some authors have highlighted certain characteristics that telesupervision would have, among them its effectiveness in establishing a specific communication style based on authentic and empathetic relationships. It would also help to reduce travel times and accessibility obstacles, allowing access to a greater number of supervisors around the world and a freer expression due to the absence of face-to-face contact [61]. They emphasize that integration between supervision and new information technologies seems an increasingly likely scenario [31]. Among the limitations, they have highlighted data latency, connection problems and signal deficiencies that would affect the alliance in monitoring [61,62], in addition to difficulties associated with Internet security and possible confidentiality issues with the data provided by patients [31]. In telehealth, in both telemedicine [63] and telepsychology [64], there are legal considerations that need to be addressed prior to implementation, including the delineation of respective prohibitions, responsibilities and competencies. Caver et al. [65] acknowledge these types of barriers in the use of remote technologies but assume that they can be overcome as there is more education and experience in their use. A few years ago, the Guidelines for the Practice of Telepsychology [66] suggested that a sufficient amount of time be allotted for in-person supervision for the required competencies to be achieved due to the preponderance of face-to-face training, while in the Standards of Accreditation for Health Service Psychology, and Accreditation Operating Procedures [67], last approved March 2022, notes that psychotherapist training programs should update and disseminate information on the use of distance education technologies for training and clinical supervision. Possibly, it is thinking about a technological era where clinical care and telesupervisions are increasingly common, in which Internet-based training methods are key to the transmission of psychotherapeutic skills [68]. Thus, part of the issues that have been identified include technology-related aspects and their consequences for practice, such as the role of asynchronous and synchronous electronic exchanges, ethical, legal, and clinical risk issues, and the competence of supervisors and supervisees in the respective technical skills in telepractice [69].

The five studies reviewed have found evidence on some of the points described above and have left other aspects uncovered. While they show that efficacy, alliance, and satisfaction in both supervision modalities (telesupervision/face-to-face supervision) appear to be similar [52,53,56], they also recognized differences in favor of face-to-face supervision [53,55,56,57] that can be considered in the area of nonverbal communication and the expression of emotions, the feeling of well-being in a shared space, and the possibility of recognizing the construction of meanings in others. It would also be pertinent to ask the question of how supervisees have experienced the construction of meaning of their supervisors in this new modality of supervision. There are aspects that can be considered on a border between the benefits and limitations of telesupervision, the challenges of a training tool and a social practice mediated by technologies of this type can favor and hinder interaction processes that traditionally took place face to face, which may help explain the transition between vulnerability and adaptation to its functioning that was observed in one of the studies included [55,56]. This transition from novelty and unfamiliarity to unfolding in different remote environments may also underlie the preference noted by some study participants for face-to-face supervision [53], even though, likewise, some participants [52] would return to having telesupervisions. From this perspective, it is also possible to understand the novelty in the ethical safeguards that are being established since the implementation of this type of supervision, which could be interpreted as a precaution of the supervised [51] regarding the private material of patients that can be shared with supervisors already aware of the risks of electronic security. 

However, another relevant question regarding telesupervision seems to be located both within and beyond its technological implementation. Wright and Griffiths [70] consider that it is necessary to study the influence of technology on distance supervision, especially in contexts where face-to-face supervision is not easy to achieve, emphasizing that supervision allows exploring and developing professional identity, influencing competence and the theory/practice linkage, as well as favoring self-care and helping to maintain an ethical practice. In the study by Perry [57] it was evidenced that both supervisors and supervisees experienced telesupervision as an effective means for the construction of professional identity. Beyond highlighting the advantages of the global approach enabled by Internet-based technology (which undoubtedly contributes to identity in terms of its potential for trans-regional, and even trans-cultural influence), and the value it rescues regarding the processes of professional identity formation, this study did not systematize the possible specific advantages that telesupervision would have in terms of the development of professional identity beyond suggesting that this may be a modality preferred by professionals born in the so-called era of “digital natives.” These and similar themes such as the transgenerational linkage between supervisor and supervisee mediated by technology could guide future research in this area.

Undoubtedly, more experience in the practice of telesupervision is needed to understand to what extent its perceived benefits and difficulties may affect its implementation and whether and what kind of consequences may influence both its “formative-instructional” and “expressive-relational” dimensions [71]. The first dimension could be clearly favored by the inclusion of new technologies, despite the problems of time lag, intermittency, handling, and electronic security, while the second would need a more careful reflection because while technical difficulties may affect the formation of professional identity also those problems with the non-verbal communicational dimension, emotional expression, and virtual representation/presentation may have effects not yet known in the supervisor/supervised interactional field and in the construction of the professional identity of the supervisees. Even more so if the dynamic influence of supervisor–supervisee interaction is considered. That is, if it is understood that supervision is a scenario in which the supervisees are active actors of their identity development as the tasks proper to the supervision space are put into practice, mediated by the presence of selves in permanent dialogue and generation of new positions [27], in addition to reconfigurations of those that were already moderately shaped, recognized, or even rigidified in both protagonists of the supervision. Finally, part of this identity process in supervisors could also be studied by observing the construction, re-construction, and/or deconstruction associated with their role comparatively in face-to-face, telesupervision, and/or mixed supervision devices.

## 5. Conclusions

This review included a bibliometric and systematic review method on the scope and limitations of virtual, digital supervision, or telesupervision in psychotherapy. In addition, it complemented and enriched its analysis based on articles published in journals indexed in the JCR-WoS, with three articles on the same topic that were already included in a previous review that did not consider the first bibliometric technique indicated in the present methodological design. In this way, it was possible to incorporate six articles that covered five original empirical studies on telesupervision and that were evaluated with a tool (MMAT) that has been applied in several reviews related to health and mental health [72,73,74] among others.

These results were based on a strict evaluation procedure and process that allowed a narrative synthesis (and discussion accordingly) despite the conceptual and methodological heterogeneity of the studies. Although one of the limitations was that this review did not consider articles outside the WoS databases (although there were some among those that were already part of the previous review), this was at the same time one of its quality guarantees. Although the final number of articles reviewed is limited, the search strategy of the review and the quality of these articles allowed for solid findings. Finally, this review accomplished its aim based on empirical studies in the field, highlighting the challenges that psychotherapists will face in their professional practice in the digital era.

## Figures and Tables

**Table 1:** Eligibility criteria using PICOS (participants, interventions, comparators, outcomes, and study design).

PICOS	Description
Participants	Professionals or professionals in training who have been supervised in the role of psychotherapist. Professionals supervising psychotherapy.
Interventions	Assignment to some form of telesupervision; application of self-report questionnaires; application of semi-structured and open-ended interviews.
Comparators	Control groups where appropriate, different modalities of supervision or other interventions, etc.
Outcomes	Results from valid and reliable measurement scales, and/or consistent with the respective reliable qualitative methods.
Study design	Qualitative designs, quantitative randomized controlled trials, quantitative nonrandomized, quantitative descriptive and mixed methods, both cross-sectional and longitudinal.

**Table 2:** Bibliometric selection by document type.

Document Types	Record Count (Percentage)
Article	793 (89%)
Proceeding Paper	45 (5%)
Review Article	23 (3%)
Early Access	20 (2%)
Book Review	15 (2%)
Total	896 (100%)

**Table 3:** Characteristics of the reviewed studies.

Authors, Year (Country)	Participants (N)	Professional Qualification	Interventions	Comparator	Outcomes	Study Design
Blackman, R., Deane, F.P., Gonsalvez, C., & Saffioti, D. (2017) [51]. (Australia)	25 participants (23 women; two men)	Seven psychologists in training; three registered psychologists and 15 registered clinical psychologists (Masters = 11 and Phd = 4)	Twenty-one-item online survey about Clinical practice behavior and perceived risk rating. Three-item online survey about Self-rated knowledge of digital security. Two-item online survey about comfort sharing recordings or notes electronically (to evaluate telesupervision).	Not applicable	Greater awareness of electronic security was inversely correlated with comfort sharing video or audio (of consenting clients) in telesupervision (rs = −0.36, *p* = 0.04).	Quantitative descriptive study
Inman, A.G., Soheilian, S.S., & Luu, L.P. (2019) [52]. (USA)	15 participants (12 women; three men)	Counseling psychology supervisors in training (Ph.D. = 7, M.Ed. = 6, M.A. = 1 and B.A. = 1).	Five open-ended questions on the challenges, benefits, ethical issues, and effectiveness of face-to-face supervision vs. telesupervision (analysis using consensus-modified qualitative research [CQR-M]) A sixth quantitatively scored question contained seven items addressing attitudes toward telesupervision.	Questions related to both types of supervision	Two-thirds of the participants thought that the quality of face-to-face supervision was better than telesupervision and one-third thought that the two formats were of equal quality. Most participants had positive attitudes toward both formats, considering them equally effective, allowing for strong supervisory relationships, with high developmental impact, and keeping on task. While one-third felt that they were more likely to be kept on task in telesupervision. Most indicated that they would participate in telesupervision again.	Convergent mixed study
Tarlow, K.R., McCord, C.E., Nelon, J.L., & Bernhard, P.A. (2020) [53]. (USA)	3 participants	Psychology doctoral students	Supervision Satisfaction Questionnaire (SSQ). Supervisory Working Alliance Inventory: Trainee Form (SWAI). Semi-structured interviews supervision vs. telesupervision experiences.	Starting with supervision and then changing to telesupervision at different times for each participant	There were no changes in supervision satisfaction and working alliance among participants in both modalities. One increased level in working alliance (𝝉 = 0.537, *p* = 0.035) when transitioning to telesupervision. Participants reported minor differences between the two modalities (difficulties perceiving nonverbal cues in telesupervision) and that effective supervisor needed to be familiar with telesupervision technology, although they preferred in-person supervision.	Case study with mixed design
* Gammon, D., Sørlie, T., Bergvik, S., & Høifødt, T.S. (1998) [55]. (Noruega)	8 participants	6 psychiatry residents and 2 supervisors	Semi-structured interviews based on communication research in social psychology and qualitative characteristics of the supervision process in psychotherapy (Content analysis not specified).	Supervisions and telesupervisions interspersed in each dyad (ABAB Design)	Participants expressed concerns (regarding telesupervision) about the reduction in nonverbal cues, and the effects these may have on spontaneity, the expression of personal emotional material, and the experience of social and emotional presence. They considered telesupervision to have positive effects (e.g., verbalization, structure, self-representation, potential as a teaching tool), which were also recognized as limitations.	Qualitative descriptive study
* Sorlie, T., Gammon, D., Bergvik, S., & Sexton, H. (1999) [56] (Noruega)	8 participants	6 psychiatry residents and 2 supervisors	Self-report questionnaire that included the dimensions: communication, contact, and supervisory alliance. The quality of supervisor-supervisee contact evaluated by means of a scale applied by external observers.	Supervisions and telesupervisions interspersed in each dyad (ABAB Design)	Supervisees scored higher on “disturbance” (frustration and displeasure) than supervisors (11.7 vs. 7.6). Supervisors scored “alliance” higher (18.8 vs. 15.1), while the overall mean score on “communication” (27.3) was identical. Supervisors experienced no significant differences in the factors “communication,” “alliance,” and “disturbance” between the two conditions. Supervisees experienced the face-to-face condition as the most favorable, especially about “disturbance” situations. Independent ratings of the video recordings revealed no difference in the variable “continuity of contact” in both supervision formats.	Quantitative descriptive study
* Perry, C.W. (2012) [57]. (USA)	16 participants (5 women; 4 men among the students)	9 students from a university clinical training program and 7 supervisors	Open-ended interviews on supervisees’ experience of professional identity (Phenomenological analysis).	Not reported	Both supervisors and supervisees experience telesupervision as an effective means for professional identity growth. Although supervisors also feel that by not having a face-to-face encounter there is no real sense of what supervisees are signifying.	Qualitative phenomenological study

* Articles originally included in the content review and analysis conducted by Inman and collaborators [54].

**Table 4:** Quality assessment of the selected studies.

Authors, Year (Country)	Category of study Designs	Methodological Quality Criteria	Responses			
			Yes	No	Cannot Tell	Comments
Blackman, R., Deane, F.P., Gonsalvez, C., & Saffioti, D. (2017) [51]. (Australia)	Screening questions (For all types)	S1. Are there clear research questions?	x			
		S2. Do the collected data allow to address the research questions?	x			
	1. Quantitative descriptive	1.1. Is the sampling strategy relevant to address the research question?	x			
		1.2. Is the sample representative of the target population?		x		
		1.3. Are the measurements appropriate?	x			
		1.4. Is the risk of nonresponse bias low?	x			
		1.5. Is the statistical analysis appropriate to answer the research question?	x			
Inman, A.G., Soheilian, S.S., & Luu, L.P. (2019) [52]. (USA)	Screening questions (For all types)	S1. Are there clear research questions?	x			
		S2. Do the collected data allow to address the research questions?	x			
	2. Mixed methods	2.1. Is there an adequate rationale for using a mixed methods design to address the research question?	x			
		2.2. Are the different components of the study effectively integrated to answer the research question?		x		
		2.3. Are the outputs of the integration of qualitative and quantitative components adequately interpreted?	x			
		2.4. Are divergences and inconsistencies between quantitative and qualitative results adequately addressed?			x	
		2.5. Do the different components of the study adhere to the quality criteria of each tradition of the methods involved?	x			
Tarlow, K.R., McCord, C.E., Nelon, J.L., & Bernhard, P.A. (2020) [53]. (USA)	Screening questions (For all types)	S1. Are there clear research questions?	x			
		S2. Do the collected data allow to address the research questions?	x			
	3. Mixed methods	3.1. Is there an adequate rationale for using a mixed methods design to address the research question?	x			
		3.2. Are the different components of the study effectively integrated to answer the research question?	x			
		3.3. Are the outputs of the integration of qualitative and quantitative components adequately interpreted?	x			
		3.4. Are divergences and inconsistencies between quantitative and qualitative results adequately addressed?		x		
		3.5. Do the different components of the study adhere to the quality criteria of each tradition of the methods involved?	x			
* Gammon, D., Sørlie, T., Bergvik, S., & Høifødt, T.S. (1998) [55]. (Norway)	Screening questions (For all types)	S1. Are there clear research questions?	x			
		S2. Do the collected data allow to address the research questions?	x			
	4. Qualitative	4.1. Is the qualitative approach appropriate to answer the research question?	x			
		4.2. Are the qualitative data collection methods adequate to address the research question?	x			
		4.3. Are the findings adequately derived from the data?	x			
		4.4. Is the interpretation of results sufficiently substantiated by data?	x			
		4.5. Is there coherence between qualitative data sources, collection, analysis, and interpretation?		x		
* Sorlie, T., Gammon, D., Bergvik, S., & Sexton, H. (1999) [56] (Norway)	Screening questions (For all types)	S1. Are there clear research questions?	x			
		S2. Do the collected data allow to address the research questions?	x			It is noted that there is a qualitative part of the study, but it has already been published in a previous article.
	5. Quantitative descriptive	5.1. Is the sampling strategy relevant to address the research question?	x			
		5.2. Is the sample representative of the target population?		x		
		5.3. Are the measurements appropriate?	x			
		5.4. Is the risk of nonresponse bias low?	x			
		5.5. Is the statistical analysis appropriate to answer the research question?	x			
* Perry, C.W. (2012) [57]. (USA)	Screening questions (For all types)	S1. Are there clear research questions?	x			
		S2. Do the collected data allow to address the research questions?	x			
	6. Qualitative	6.1. Is the qualitative approach appropriate to answer the research question?	x			
		6.2. Are the qualitative data collection methods adequate to address the research question?	x			
		6.3. Are the findings adequately derived from the data?	x			
		6.4. Is the interpretation of results sufficiently substantiated by data?		x		
		6.5. Is there coherence between qualitative data sources, collection, analysis and interpretation?		x		

* Articles originally included in the content review and analysis conducted by Inman and collaborators [54].

## Data Availability

The data presented in this study are available in Supplementary file.

## Supplementary Materials

The following supporting information can be downloaded at: https://www.mdpi.com/article/10.3390/ijerph192316366/s1. Supplementary File S1. The supplementary file “Web of Science records” presents details of the 396 pre-selected articles, according to the data provided by Clarivate Analytics Web of Science. It can be opened with VOSviewer or other specialized bibliometric software.
